# Supplementary material for: Systematic Analysis of the Grafting-Related Glucanase-Encoding GH9 Family Genes in Pepper, Tomato and Tobacco
Source: Plants (Basel). 2022 Aug 11;11(16):2092. doi: 10.3390/plants11162092 (PMC9414958; doi:10.3390/plants11162092)
Supplement: Supplementary file 1 [file plants-11-02092-s001.zip › Figure S1.pdf]

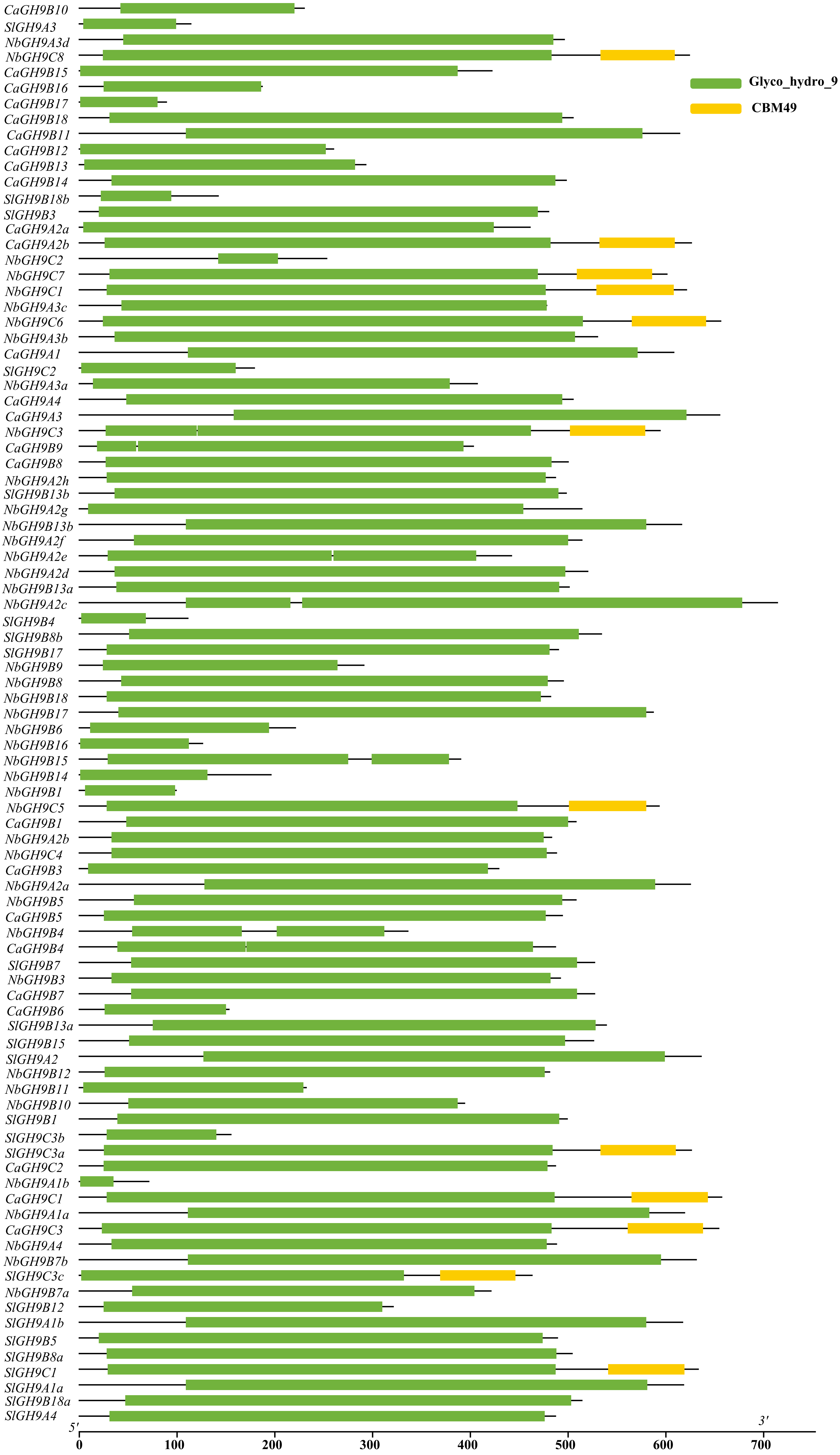

Figure S1. Protein domains of CaGH9, SlGH9 and NbGH9 family members.
Green and yellow block represents the Glyco_hydro_9 and CBM49 domain, respectively.
